# Coalescence of Immiscible Liquid Metal Drop on Graphene

**DOI:** 10.1038/srep34074

**Published:** 2016-09-26

**Authors:** Tao Li, Jie Li, Long Wang, Yunrui Duan, Hui Li

**Affiliations:** 1 Key Laboratory for Liquid-Solid Structural Evolution and Processing of Materials, Ministry of Education, Shandong University, Jinan 250061, People’s Republic of China

## Abstract

Molecular dynamics simulations were performed to investigate the wetting and coalescence of liquid Al and Pb drops on four carbon-based substrates. We highlight the importance of the microstructure and surface topography of substrates in the coalescence process. Our results show that the effect of substrate on coalescence is achieved by changing the wettability of the Pb metal. Additionally, we determine the critical distance between nonadjacent Al and Pb films required for coalescence. These findings improve our understanding of the coalescence of immiscible liquid metals at the atomistic level.

The immiscible alloys are well-known and widely used because of their excellent mechanical, magnetic, and thermal properties^1^,^2^,^3^. For instance, Al-Pb, Al-In and Al-Bi alloys are used for the fabrication of porous aluminum with pores at micrometer size^4^. Al-Pb alloys have been developed to make bearings in car engines that exhibit less wear and demonstrate improved load-bearing capabilities^5^. However, liquid Al and Pb are not mutually soluble in liquid phase^6^, which may restrict full application of this alloy. Because of this limitation, it is important to determine whether or not liquid Al and Pb drops can coalesce in the immiscible region and understand the behavior of coalescence. Coalescence is a key process in nature by which two or more droplets or bubbles merge during contact to form a single big droplet or bubble, so coalescence may play a significant role in the metallurgical process. Coalescence takes place in both natural and technological processes, including raindrop formation in clouds^7^, inkjet printing^8^,^9^, spray coating^10^, microfluidic devices^11^, and filtration^12^,^13^. During spray coating, the perfect merging of drops is critical to the quality of the resulting solid coating.

Numerous theoretical or experimental studies have been performed to investigate the dynamics of coalescence^14^,^15^, for both viscous^16^ and low-viscosity liquids^17^. The dynamics of coalescence on substrates consists of two stages: the rapid growth of a liquid bridge between films in the early-time stage, and a second stage in which the merged droplet shape changes from elliptical to circular. Early work on coalescence mainly focused on the latter stage using sessile drops^14^,^15^,^18^, but the kinetics of the first stage is of significant interest for industrial and biochemical applications^19^. During this inital stage, Karpitschka *et al*.^20^ reported that sessile droplets instantaneously fuse upon contact at their three-phase lines due to the capillarity force and a surface tension gradient. The width *d*_*0*_of the meniscus bridge between two drops (with viscosity *μ*, surface tension *γ*.radii *R*_*0*_ and heights *h*_*0*_) is governed by a scaling law^16^, 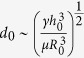. Additionally, the growth of bridge occurs according to a geometric model obeying the scaling law with 2/3 or 1/2 exponent by Eddi 21. Although much work has been done to study the dynamics of droplets coalescence, existing studies have primarily focused on two drops of the same material, and the coalescence behavior of metal films with different wettability during the early stage remains obscure, particularly for immiscible alloy film. Furthermore, whether and how the substrate affects the wettability of two metals is poorly understood.

Graphene is often used as a crucible material that can be used for alloy smelting. Additionally, graphene becomes popular for other applications due to its exceptional physical performance, such as its use in graphene transistors^22^,^23^,^24^,^25^, all-graphene-battery^26^, and supercapacitor devices^27^,^28^. In this work, we performed molecular dynamic (MD) simulations to explore the coalescence behavior of Al and Pb films on different graphenes at the nanoscale and discovered the effect of substrates on the behavior of the two metals during alloy formation, which may provide theoretical guidance for fabricating this alloy. Besides, we discuss the variation of wettability for different substrates, which will be useful for applications in metallurgy^29^.

## Results

**Figure 1:**
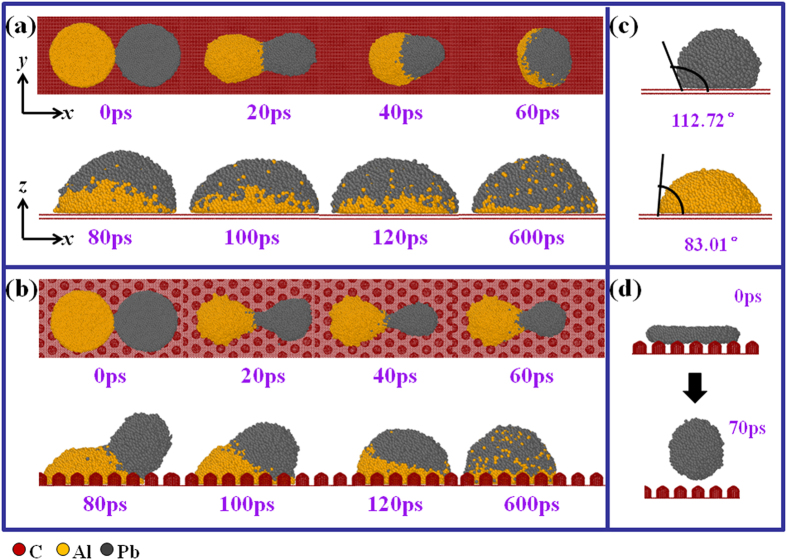
Cocalescing process of Al and Pb films in contact with (**a**) double-wall graphene (DG) and (**b**)pillared graphene (PG) surface (gray atoms indicate Pb atoms, yellow atoms indicate Al, and red atoms indicate the carbon-based substrate; coloring is the same in the following figures). The drop-forming time is indicated in the figure. It takes a longer time for the two circle films to form a new spherical drop on PG. (**c**) Singular Al and Pb film on DG at 300 ps. The wetting contact angle of Pb is larger than 90° but it is smaller than 90° for liquid Al after reaching the equilibrium state, implying the wetting of liquid Al on graphene is stronger than liquid Pb. (**d**) Dewetting behavior of Pb film on PG. The droplet detaches from the PG surface after 70 ps.

The early-time coalescence evolution of Al and Pb films on DG and PG substrates is presented in Fig. 1. Initially, the two liquid films are adjacent to each other and are located on the graphene sheet. As the simulation starts, these two immiscible films tend to coalesce at a high speed and finally convert into one droplet. Interestingly, the droplet forming time is quite different between two substrates. About 60 ps is required to form a new spherical drop on DG as shown in Fig. 1(a). Compared with DG, the coalescing time on PG is approximately twice of the DG, suggesting that PG does not promote the merging. The side–view images of the coalescence process are shown to explain the difference in coalescing time on two substrates. Apparently, on DG, atoms merge into each other instantly in the *x* direction. However, on PG, Pb atoms exhibit a strongly upward motion (*z* direction) and detach from the substrate, and then the Pb drop begins to blend with the Al drop along the *x* direction. To better understand the different behavior of two drops on DG and PG, the wettability of Pb films on these two substrates was also explored. Figure 1(c) shows that the wetting contact angle of Pb is larger than 90° but it is less than 90 ° for liquid Al, revealing that the wettability of Pb on double graphene is weaker than Al. However, both of them still attach to the DG surface. When applied to the surface containing some microconvexities or greater roughness as shown in Fig. 1(d), the Pb drop can detach from the surface indicating its dewetting property^30^,^31^. Liquid Al penetrates the interspaces of pillared nanotubes, and does not display dewetting behavior. From this, we can conclude that the different wetting of Pb on DG and PG results in the different coalescing behavior.

**Figure 2:**
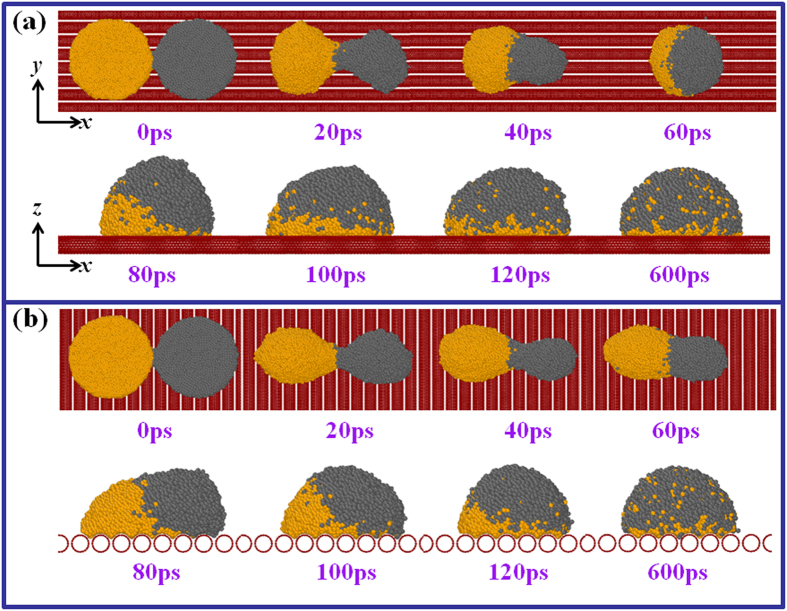
Coalescing process of Al and Pb films on (**a**) horizontally-placed carbon nanotubes (HCNT) and (**b**) vertically-placed carbon nanotubes (VCNT) surface. The time for obtaining a larger droplet is different for HCNT and VCNT.

Figure 2 shows the coalescing process of two films on CNTs substrates. It is notable that the formation of a new spherical drop on HCNT needs about 60 ps, but 100 ps is required to form one drop on VCNT, longer than that on HCNT. Clearly, the direction of the substrate groove has a strong influence on the motion of atoms. The vertical carbon nanotubes go against the diffusion of the atoms along *x* direction, increasing the time required to complete the coalescence.

**Figure 3:**
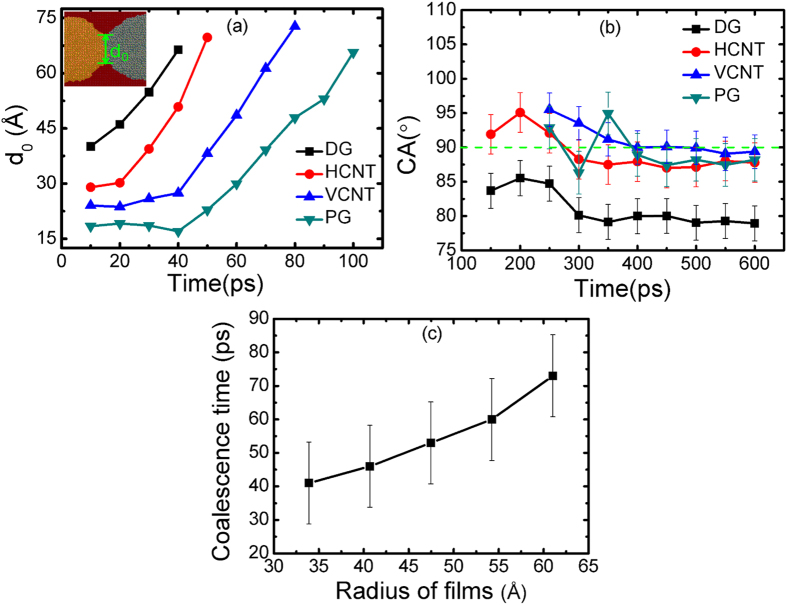
(**a**) Width d_0_ of liquid bridge as a function of time. (**b**) Contact angles (CAs) on the four substrates with error bars of standard deviation (the dash in the graph indicates the 90°), which is measured by averaging five simulations. The CAs show very little change after 400 ps. At 600 ps, the CAs on DG, HCNT, VCNT, and PG are 78.94°, 87.79°, 89.37° and 88.20°, respectively. (**c**) Coalescence time (average of five values) on DG at the varying radius of films with error bars of standard deviation.

During the coalescing course, a liquid bridge forms between two drops, but the growing rate of width is different on the four substrates. As presented in Fig. 3(a), the width of the growing meniscus bridge d_0_ is more likely to show a linear increase (after the d_0_ begins to increase) on the four substrates, which does not obey the scaling law^16^. As we mentioned above, the moving behavior of the Pb drop on the four substrates determines the coalescence time, so the width is primarily relevant to the Pb drop instead of the two drops. This may be the reason why d_0_ does not obey this law. Before 40 ps, the width on DG surface is the thickest, followed by that on CNTs, and then the PG surface. On DG and HCNT substrates, the width increases rapidly with time, but on the VCNT and PG, the width hardly changes before 40 ps. After 40 ps, the curves also exhibit a rapid nearly-linear increase on VCNT and PG. So when we compare the HCNT with VCNT, it is obvious that the direction of nanotubes affects the growth rate of the liquid bridge. Although the bridge width on PG appears thinner before 40 ps, the films finally turn into a whole drop, implying that the liquid film aims to reduce the surface tension by coalescence. Additionally, there is a strong interaction between the Al and Pb atoms, considered a driving force for contraction.

The contact angle (CA) of the liquid droplet on a substrate is predominantly used to characterize the surface wetting properties, and here was measured by averaging simulations performed five times. The CA is the angle between the substrate and the tangent to the particle surface starting from the triple point. As shown in Fig. 3(b), the contact angle of Al-Pb alloy at 600 ps is quite different for the four substrates, for a CA on DG, HCNT, VCNT, and PG of 78.94°, 87.79°, 89.37°, and 88.20°, respectively. The gap between the maximum and minimum CA on CNTs and PG is less than 5°, but these contact angles are much larger than the ones on DG, suggesting that the wettability depends on the anisotropic surface topography. In conclusion, Al-Pb alloy is more likely to adsorb on the smooth surface. All the CAs are less than 90°, indicating that the coalesced liquid alloy exhibits good wetting. This may be responsible for the good wettability of Al on graphene and the observed separation of Pb from the surface. When two immiscible metal drops with different wetting properties coalesce, the wetting ability of the coalesced drop is related only to that of the wetting metal instead of the non-wetting metal. Fig. 3(c) shows the coalescence time at the varying radius of films. The coalescence time showed a rapid non-linear increasing trend with particle size, manifesting that the particle size can affect the coalescence dynamics.

**Figure 4:**
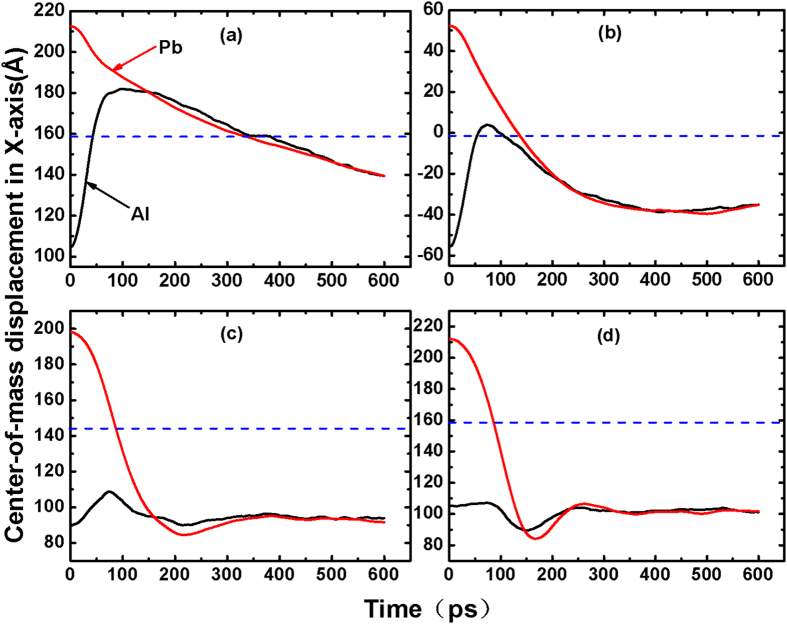
Center-of-mass displacement of the Al and Pb atoms along the *x* direction as a function of time. The dashed line represents the middle position between the Al and Pb films. The *x* direction corresponds with the X-axis in Figs 1 and 2. (**a**) DG, (**b**) HCNT, (**c**) VCNT, and (**d**) PG.

Figure 4 shows the center-of-mass of the Al and Pb drops along the *x* direction (CMD_*X*_) as a function of time. The dash in the figure represents the middle position between the Al and Pb films, which is the boundary between the Al and the Pb drop. From Fig. 4(a–d), all Pb drops finally approach the Al on the four substrates after 600 ps, suggesting that Pb atoms have a large displacement. Because weaker interaction between Pb and carbon decreases the wettability of Pb compared to Al, the movement of Pb atoms is not impeded by the substrate. It is found that the CMD_*X*_ of Al (Al-CMD_*X*_) exhibited the largest peak on DG, followed by CNTs, but there was a valley on PG. Due to the interaction between Al and Pb, Al and Pb atoms move towards each other. Al atoms have limited movement in the right direction of the X-axis because of the restriction from Pb atoms. On DG, the surface is smooth, so the Al drop has a greater tendency to move towards Pb with the driving force, leading to a larger peak of Al-CMD_*X*_. On CNTs, although the surface is rough, the Al-Pb interaction is still greater than the force of friction, resulting in a smaller peak of the Al-CMD_*X*_. However, on PG, the Al atoms can penetrate the interspaces of the pillared nanotubes and fix on the surface as shown in Fig. 1(b). Because of this pinning effects caused by pillared nanotubes, the movement of the Al is restricted, hindering its ability to move close to the Pb drop. Later, when the two metallic drops become one larger drop, they begin to move to the left in the X-axis direction because of the inertia force caused by the Pb atoms. Thus, a valley forms in the Al-CMD_*X*_. With the curves close to each other, two droplets become one larger drop. Overall, the substrate can significantly affect the movement of atoms to determine the coalescence behavior.

**Figure 5:**
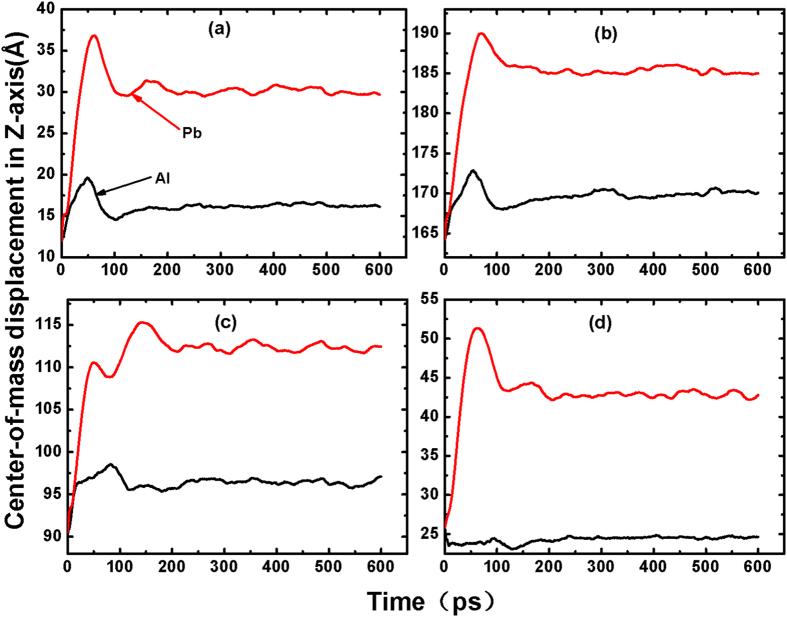
Center-of-mass displacement of the Al and Pb atoms along the *z* direction as a function of time. (**a**) DG, (**b**) HCNT, (**c**) VCNT, and (**d**) PG. The *z* direction corresponds with the Z-axis in Figs 1 and 2. Pb atoms are above the Al atoms with a large distance.

Figure 5 shows the center-of-mass of the Al drop and the Pb drop along the *z* direction (CMD_*Z*_) as a function of time. In the beginning, Pb atoms exhibit a strong upward motion on the four substrates, giving rise to a big gap between Al- and Pb-CMD_*Z*_. All the Pb-CMD_*Z*_ show a peak, implying that there is a distance limitation for atoms to detach from the surface due to the better wettability of Al on graphene. However, the distance between the Al peak and the Pb peak on the same substrate is quite different. It is about 17 Å on DG and HCNT, about 18 Å on VCNT, and almost 29 Å on PG. Obviously the PG substrate has the biggest influence on the motion of atoms. When the two lines are approximately parallel, the two droplets become one drop.

**Figure 6:**
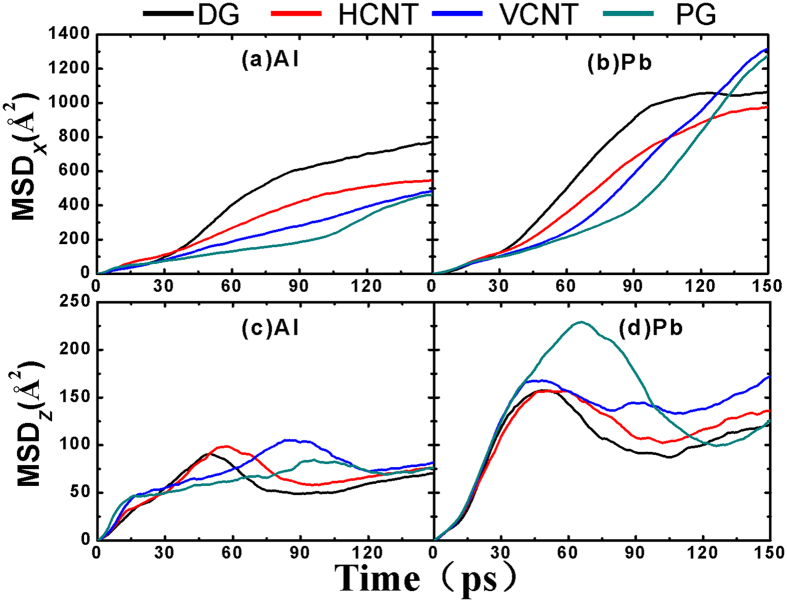
Time-evolution mean square displacement (MSD) of Al and Pb atoms in different directions. The MSD of (**a**) Al atoms and (**b**) Pb atoms in the *x* direction. The MSD of (**c**) Al atoms and (**d**) Pb atoms in the *z* direction.

The diffusivity of the atoms, considered as another physical quantity to describe the behavior of liquid metals on the substrate, is very important. So the time-evolution mean square displacement (MSD) of Al and Pb atoms is shown in Fig. 6 to illuminate the movement of atoms in the *x* and *z* directions during coalescence before 150 ps. Due to the different wettability, the diffusion speed of Al atoms is much smaller than that of the Pb atoms in both *x* and *z* directions. Furthermore, for both Al and Pb atoms, the diffusion speed in the *x* direction is greater than that in the *z* direction, suggesting that the metal-carbon interaction may play a partial role in hindering the diffusion of atoms along the *z* direction. In contrast, the Al-Pb interaction can promote the diffusion of atoms along the *x* direction. It is worth mentioning that the slope of the MSD_*x*_ curve for Al and Pb atoms on PG is smaller than that on the other substrates, but the slope of MSD_*z*_ curve for Pb on PG is the largest, indicating increased time for the coalescence on PG. At longer simulation time, the MSD_*x*_ slope of Pb on VCNT and PG increase faster than that on DG and HCNT, resulting from the larger upward movement of Pb atoms as shown in Fig. 6(d). Therefore, the diffusion speed increases faster along the *x* direction with the weaker effect of substrates. Finally, the different diffusion speed between HCNT and VCNT provides further evidence to illuminate the different time required to complete the coalescence.

To determine the effect of substrate on the self-diffusion coefficient of Al and Pb atoms, we calculated the self-diffusion coefficients of Al and Pb after reaching equilibrium (from 400 ps to 600 ps). The self-diffusion coefficient (D) can be derived from the mean square displacement (MSD)−time curve according to the Einstein diffusion law:


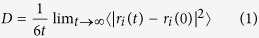


Where *r*_*i*_*(t)* is the position of atom *i* at time *t* and 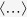 denotes an average overall atoms^32^. To minimize the influence of other factors, we selected the metal-DG system. According to the Einstein diffusion law, the D of Al and Pb is 0.473 Å^2^/ps and 0.223 Å^2^/ps, respectively. In other experiment or simulation works, the D of Al was determined as 0.52–0.68 Å^2^/ps (943 K–1323 K)^33^, and the D of Pb was calculated as 0.168 Å^2^/ps (600 K)^34^. Due to the small effect of substrate and the higher temperature (1500 K), the D of Pb should be larger in our works. Additionally, the stronger interaction between aluminum and substrate should cause the D of Al to be smaller than other systems.

**Figure 7:**
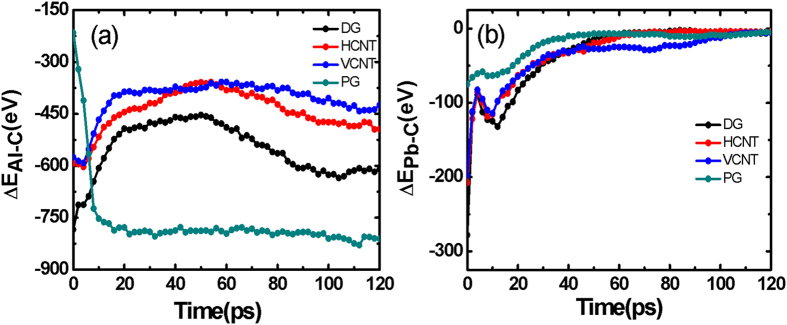
Interaction energy. (**a**) The variation of aluminum-carbon interaction energy ∆E_Al−C_ versus time. (**b**) The variation of plumbum-carbon interaction energy ∆E_Pb−C_ as a function of time.

Why is the initial diffusion speed of Pb atoms greater than that of Al atoms? One key factor in determining the motion of atoms is the interaction energy between metal and carbon, as the larger the interaction energy, the lower the diffusion speed. To find out the reasons for this, we plotted the energy-time curves as shown in Fig. 7(a,b). *ΔE* is given as:


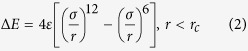


Where ε is the depth of the potential wall, σ is the finite distance at which the inter-particle potential is zero, and *r*_*c*_ is the cutoff distance. The Pb-C interaction energy is weaker, and gradually decreases to zero after 100 ps, implying that the Pb atoms totally detach from the graphene surface. However, the Al metals still remain in contact with the substrate because of the strong interaction energy that restricts the diffusion of Al atoms in the *x* and *z* directions. Interestingly, the increasing of the Al-C interaction energy on the PG surface shown in Fig. 7(a), is different from that of other substrates, since Al atoms penetrate the interspaces of the pillared nanotubes as shown in Fig. 1(b). As a result, the diffusion speed of Pb atoms is higher than Al atoms.

**Figure 8:**
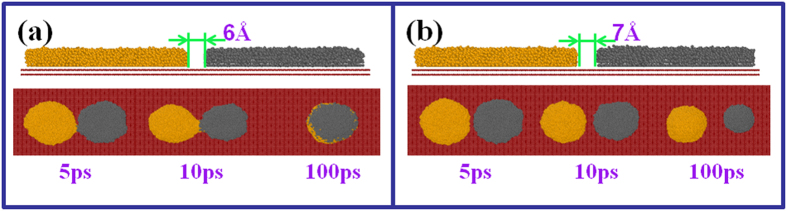
Different spacings between Al and Pb films before coalescing and snapshots of the coalescing process from 5 ps to 100 ps. (**a**) 6 Å. (**b**) 7 Å.

Two liquid drops can coalesce into a bigger drop by forming a liquid bridge when they initially contact each other. What distance between two liquid drops is required to form this liquid bridge? We next performed serial simulations to calculate this critical distance. Figure 8 gives the different distances between Al and Pb films on DG before coalescing. The liquid bridge can form if the distance is less than 6 Å, but if the distance is 7 Å or larger, bridging does not occur and two independent drops are obtained ultimately. Thus, the critical distance for films to merge on DG is 6 Å. Simulation results show that not only the contacting distance, but also the interaction between the two liquid drops determines whether or not the coalescence can occur. Similar simulations have been performed on CNTs and PG as shown in Table 1. The critical distance is 4 Å on HCNT and 3 Å on VCNT and PG, less than that on DG, indicating that the critical distance is also determined by the surface microstructure.

## Discussion

In summary, the microstructure and surface topography of substrate both affect the coalescence behavior of metal films via transforming the weak wetting on DG into non-wetting on PG for Pb metal in the early-time stage. This is evident by the length of coalesced time, the growth rate of the liquid bridge and the speed of diffusion. Different coalescing behaviors on CNTs further demonstrate the significant role of substrate. Additionally, films can still merge into a lager droplet at certain spacing distance between films, but this critical spacing distance is also influenced by the surface microstructure and therefore is different for the four substrates. Our findings offer a better understanding of coalescence at the atomic level and provide an efficient method to tune coalescence by alteration of the wetting of materials, allowing promising applications in spray coating or in microfluidic devices.

## Methods

In this paper, MD simulations were performed to study the coalescence of liquid Al and Pb drops on the double-wall graphene (DG), pillared graphene (PG), horizontally-placed carbon nanotubes (HCNT) and vertically-placed carbon nanotubes (VCNT). To improve the computational efficiency, all the substrates were fixed during simulation progress. Periodic boundary conditions are applied in the *x, y* and *z* directions, and the simulation box size used is 39.1 × 24.7 × 35.9 nm^3^.

Circular Al and Pb films with the size of 15 Å in thickness and the size of 54.12 Å in radii, were obtained by melting pure metal at 1500 K and relaxing for 500 ps. Both films were deposited at a distance of 2.0 Å above the substrate with the center connecting line of each film parallel to the substrate, at an initial distance between films of 0, then all MD simulations were run by 600 ps to study the coalescence process. Al-Al, Pb-Pb and Al-Pb interactions are described by an embedded atom method (EAM) potential^35^,^36^, which can be written as:


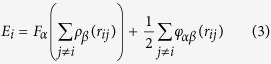


Where *F* is the embedding energy, *φ* is a pair potential interaction. The C-C interaction is modeled by an adaptive intermolecular reactive empirical bond order (AIREBO) potential^37^. Because metal and carbon can only form soft bonds via charge transfer from the π electrons in the sp^2^ hybridized carbon to the empty 4 s states of metal^38^, we utilized the 12-6 Lennard-Jones (L-J) potential with a well depth ε = 0.0309 eV and size parameter σ = 3.422 Å to describe the Al-C interactions^39^, and a well depth ε = 0.01751 eV and size parameter σ = 3.288 Å was determined by Lorentz-Berthelot combining rules^40^,^41^ to calculate the Pb-C interactions^42^. With these parameters, we calculated the equilibrium contact angle as 83.43° for Al and 112.12° for Pb on the flat graphene at 1500 K, which accord well with the experimentally measured contact angle of Al (about 85°)^29^ and Pb (about 110°)^43^.

The MD simulations were carried out using the large-scale atomic/molecular massively parallel simulator (LAMMPS) package in the NVT ensemble (the number of particles N, volume V, and temperature T were kept constant). The temperature was held at 1500 K as controlled by the Nose–Hoover thermostat^44^,^45^. The time integration of Newton’s equation of motion was calculated by the velocity Verlet algorithm^46^ with a time step of 1.0 fs.

## Additional Information

**How to cite this article**: Li, T. *et al*. Coalescence of Immiscible Liquid Metal Drop on Graphene. *Sci. Rep.* **6**, 34074; doi: 10.1038/srep34074 (2016).

## Figures and Tables

**Table 1:** Spacings between two films on four substrates, **√** stands for coalescence for two metallic films, **×** stands for non-coalescence for two metallic films.

Distance(Å)	DG	HCNT	VCNT	PG
1	**√**	**√**	**√**	**√**
2	**√**	**√**	**√**	**√**
3	**√**	**√**	**√**	**√**
4	**√**	**√**	**×**	**×**
5	**√**	**×**	**×**	**×**
6	**√**	**×**	**×**	**×**
7	**×**	**×**	**×**	**×**
